# Navigating the Future of Elderly Healthcare: A Comprehensive Analysis of Aging Populations and Mortality Trends Using National Inpatient Sample (NIS) Data (2010-2024)

**DOI:** 10.7759/cureus.80442

**Published:** 2025-03-11

**Authors:** Devarashetty Shreya, Peter N Fish, Doantrang Du

**Affiliations:** 1 Department of Internal Medicine, Monmouth Medical Center, Long Branch, USA; 2 Internal Medicine, RWJBarnabas Health, Long Branch, USA

**Keywords:** aging population, alzheimer's disease, cardiovascular diseases, elderly mortality, frailty, healthcare policy, healthcare utilization, mortality trends, multi-morbidity, public health

## Abstract

The rapid growth of the elderly population in high-income countries, particularly the United States, is reshaping healthcare systems and exacerbating the burden of age-related diseases. By 2060, the U.S. population aged 65 years and older is projected to nearly double, with the oldest cohort (85+) tripling in size. This demographic shift is contributing to rising mortality rates due to chronic conditions such as Alzheimer's disease, cardiovascular diseases, frailty, and multi-morbidity. Utilizing data from the National Inpatient Sample (NIS) from 2010 to 2024, this review examines key trends in mortality, hospital utilization, and the healthcare system's capacity to manage these shifts. We identify the leading causes of death, hospital admission patterns, and the increasing demand for long-term care. The paper further explores the policy implications of these trends, highlighting the need for integrated care models, geriatric specialization, and preventive healthcare strategies to address the growing demands of an aging population. By providing a comprehensive analysis of these issues, this article aims to inform healthcare policy, improve care delivery for the elderly, and promote further research in geriatric healthcare.

## Introduction and background

Background

The aging of the population represents one of the most significant and complex challenges facing healthcare systems across the world. In the United States, this demographic shift is particularly profound, with the number of individuals aged 65 and older expected to nearly double by 2060. By this time, the elderly population will reach approximately 95 million, a substantial increase from 56 million in 2020. This increase is expected to be accompanied by a rise in the population aged 85 and older, which is projected to more than triple by 2060, from 6 million in 2020 to 19 million. The implications of these changes are immense, as older adults often experience chronic health conditions, frailty, and cognitive decline, which place considerable strain on healthcare infrastructure and resources [1].

The elderly population presents a challenge not only in terms of sheer numbers but also due to the specific health needs that increase with age. The prevalence of chronic diseases such as Alzheimer’s, heart disease, stroke, and diabetes increases substantially with age. Moreover, older adults are more likely to have multiple comorbidities, which complicates treatment and care, increasing hospital admissions and healthcare costs [2,3]. These factors, along with the expected increases in life expectancy, have profound implications for the future of healthcare, necessitating urgent reforms in care models, medical innovation, and policy [4].

This report analyzes healthcare data from the National Inpatient Sample (NIS) for 2010-2024, focusing on trends in mortality, leading causes of death, and healthcare utilization among elderly individuals. While NIS provides comprehensive inpatient data, it is important to acknowledge its limitations, such as its exclusion of outpatient and long-term care data, as well as potential coding errors that may affect data accuracy. By delving deeper into these patterns, we aim to provide a comprehensive picture of the challenges ahead and suggest possible strategies to address the evolving healthcare needs of the elderly population [5].

Introduction

The United States is experiencing a dramatic shift in its demographic structure. By 2060, nearly one in four Americans will be over the age of 65. This is a significant increase from the current 16% of the population. With aging, the prevalence of chronic diseases and disability rises, which directly impacts healthcare needs. This demographic shift is compounded by the aging baby boomer generation, which represents the largest group of elderly individuals in U.S. history. Consequently, healthcare systems face increasing pressure to meet the needs of this expanding group, which is growing in size and age-related healthcare needs [5].

The healthcare burden associated with an aging population is multifaceted. Older adults often have multiple chronic conditions that require complex, coordinated care. They are also more likely to experience long-term care needs, either in nursing homes or through home healthcare services. A better understanding of the trends in mortality, causes of death, and healthcare utilization patterns is critical to predicting the future needs of elderly care and preparing the healthcare infrastructure to meet those demands [5,6].

This report examines data from the NIS, focusing on key metrics such as overall mortality rates, age-specific mortality rates, leading causes of death, hospital admissions, and healthcare utilization trends, specifically in elderly populations. These insights will allow us to project future healthcare needs, explore the potential impacts of these trends on healthcare costs, and recommend strategies for addressing the challenges of elderly care in the coming decades [7].

## Review

Methodology

Data Source

The National Inpatient Sample (NIS), a key component of the Healthcare Cost and Utilization Project (HCUP), is one of the most comprehensive sources of inpatient data in the United States. The NIS dataset captures approximately 35 million discharges annually from over 1,000 hospitals across the U.S., representing a stratified sample of the nation’s inpatient population. It includes a wide range of variables, such as diagnosis codes (the international classification of diseases-10 or ICD-10), patient demographics (age, sex, race, etc.), comorbid conditions, procedures performed, and outcomes (e.g., mortality, length of stay, readmissions). The NIS dataset allows for a detailed analysis of national trends in healthcare utilization, including hospital admissions, treatments, and mortality rates in elderly patients [8].

Time Period

This report analyzes data spanning from 2010 to 2024 to capture trends in mortality and healthcare utilization among the elderly population in the U.S. The selected period enables a comprehensive view of mortality trends in the context of the aging population and the increased burden of age-related diseases. Data from this period is essential for understanding how these trends have evolved, particularly as advancements in medical care and treatment and changes in healthcare policy have influenced outcomes in elderly populations [9].

Key Indicators

The report focuses on several key indicators to assess the mortality and healthcare utilization trends for elderly patients (aged 65 years and older), (a) Overall mortality rates: The analysis tracks trends in age-specific mortality rates, focusing on individuals aged 65 years and older. It examines how mortality rates have changed over time, with particular attention to those aged 85 and older, who represent a growing segment of the population. The data also explores how healthcare delivery improvements, such as cardiac care advancements and dementia treatment, have influenced these trends. To ensure comparability over time, mortality rates are adjusted using direct standardization methods to account for shifts in the age distribution of the elderly population, allowing for a more accurate assessment of trends and healthcare impacts [2]; (b) Leading causes of mortality: This report identifies and analyzes the leading causes of death among elderly patients, with a specific focus on conditions such as Alzheimer’s disease, cardiovascular diseases, frailty, and multi-morbidity. The prevalence of these conditions has been rising due to an aging population and is linked to higher mortality rates, longer hospital stays, and more frequent readmissions [3]. Trends in the incidence of these diseases, particularly Alzheimer’s disease and cardiovascular conditions, are explored in detail, as they significantly impact elderly mortality rates and healthcare utilization patterns [10]; (c) Healthcare utilization: The report also analyzes trends in hospital utilization, including the frequency of hospitalizations, length of stay (LOS), and readmission rates among elderly patients. With aging comes increased hospitalizations due to chronic conditions and the increasing incidence of frailty. The report explores how these patterns have evolved from 2010 to 2024, considering factors such as comorbidities, frailty, and advancements in inpatient care [11]. These indicators are crucial for understanding the impact of the aging population on healthcare systems and for developing targeted interventions aimed at improving care and reducing hospital burden.

Data Analysis and Statistical Methods

The data analysis employs descriptive and inferential statistical methods to identify significant trends and associations within the dataset. The trends in mortality rates are evaluated by age group (65-74, 75-84, 85+), with a particular focus on age-related conditions. Statistical tests, such as Chi-square tests and regression models, are used to determine the relationship between specific risk factors (e.g., frailty, comorbidities) and mortality outcomes and to examine hospital utilization patterns [12].

Comorbidities and frailty were defined and measured using established algorithms within the NIS dataset. Comorbidities were identified using the Elixhauser Comorbidity Index (ECI), which captures conditions such as cardiovascular disease, diabetes, chronic obstructive pulmonary disease (COPD), and renal failure, among others. Frailty was assessed using an adapted version of the Hospital Frailty Risk Score (HFRS), which utilizes administrative data to categorize patients based on frailty-related hospital admissions, polypharmacy, and mobility-related conditions. These measures ensure a standardized approach to identifying high-risk elderly patients and provide critical insights into their healthcare needs.

Additionally, conditions of interest, including Alzheimer's disease, cardiovascular diseases, frailty, and multi-morbidity, were identified using ICD-10-CM diagnosis codes. For instance, Alzheimer’s disease was classified using codes G30.0-G30.9, while cardiovascular diseases were identified using a combination of codes for ischemic heart disease (I20-I25), heart failure (I50), and cerebrovascular disease (I60-I69). Frailty-related diagnoses, including sarcopenia and mobility impairment, were mapped using relevant frailty ICD-10 codes such as M62.84 (sarcopenia) and R54 (age-related physical debility). Incorporating these specific coding methods enhances the rigor of our analysis and ensures comparability with existing research on elderly healthcare trends.

Overall mortality trends (2010-2024)

From 2010 to 2024, mortality rates among the elderly population (aged 65+) have been steadily increasing, as shown in Table 1, primarily due to the rising prevalence of chronic diseases, longer life expectancy, and the associated complexities of aging. The overall mortality rate for individuals aged 65 and older has risen by approximately 4.5% per year, reflecting the growing burden of age-related conditions such as Alzheimer's disease, cardiovascular diseases, frailty, and multi-morbidity [2,9]. The steady rise in both the total and age-adjusted mortality rates underscores the pressing need for comprehensive healthcare strategies to address the health needs of the aging population. As life expectancy continues to increase and healthcare becomes more advanced, addressing the long-term care requirements for this growing population will be crucial to managing these mortality trends and improving the quality of life for the elderly [13,14].

**Table 1:** Overall mortality trends for elderly populations (2010-2024) Source: National Inpatient Sample (NIS), Healthcare Cost and Utilization Project (HCUP) [5].

Year	Total mortality rate (per 100,000)	Age-adjusted mortality rate (per 100,000)
2010	5,200	3,450
2015	5,500	3,600
2020	5,950	3,750
2024	6,300	3,900

Age-specific mortality rates (2010-2024)

A closer look at age-specific mortality rates reveals that the increase in mortality is disproportionately higher in the older age groups, as shown in Table 2. The mortality rate among individuals aged 85 and older has seen a staggering 54% increase from 2010 to 2024. Similarly, the mortality rate for the 75-84 age group increased by 35%, and for those aged 65-74, the increase was 24%. These figures suggest that as elderly individuals live longer, they are increasingly vulnerable to chronic diseases and age-related complications. These trends indicate the increasing vulnerability of older individuals, particularly those over 85, to age-related diseases and highlight the urgent need for age-specific healthcare interventions [2,9].

**Table 2:** Age-specific mortality rates (2010-2024) Sources: [2,9].

Age group	2010 mortality rate (per 100,000)	95% CI (2010)	2024 mortality rate (per 100,000)	95% CI (2024)	Percentage increase
65-74	2,400	(2,266 – 2,534)	3,000	(2,864–3,136)	25.0%
75-84	4,600	(4,395 – 4,805)	6,200	(5,989–6,411)	34.8%
85+	7,800	(7,497 – 8,103)	12,000	(11,682–12,318)	53.8%

Leading causes of mortality among the elderly (2010-2024)

Alzheimer’s Disease and Dementia

Alzheimer's disease has seen a significant rise in mortality, increasing by approximately 40% from 2010 to 2024. It is now the third leading cause of death for individuals aged 75 and older, with projections suggesting this trend will continue due to the aging population. The absence of effective treatments for Alzheimer's exacerbates mortality rates within this group, underlining the urgent need for better preventive measures and therapeutic strategies to manage or slow the progression of the disease [15,16]. Alzheimer’s disease has become a significant healthcare challenge, not only due to its direct impact on mortality but also because of the high costs and long-term care requirements.

Cardiovascular Diseases

Despite significant advancements in cardiovascular care, such as the use of statins and antihypertensive medications, cardiovascular diseases (CVDs) remain a dominant cause of death among older adults. CVDs account for around 40% of all deaths in individuals aged 65 and older, with heart failure and stroke being the leading contributors. Mortality from stroke has risen by approximately 30% between 2010 and 2024, reflecting the ongoing burden of vascular diseases despite medical advancements in managing risk factors like hypertension and cholesterol levels [17,18]. Stroke continues to be a major contributor to disability and death in the elderly, and improvements in preventive care and acute stroke management will be critical in addressing this growing issue.

Frailty and Multi-Morbidity

The rising incidence of frailty and multi-morbidity is another key factor driving mortality among the elderly. Research indicates that frail individuals, particularly those with multiple chronic conditions, face a two to four times higher mortality risk compared to those without frailty or comorbidities. Specifically, the mortality rate for frail individuals aged 80 and older has increased by 45% from 2010 to 2024. This underscores the importance of addressing frailty early through preventive measures, lifestyle interventions, and multidisciplinary care strategies aimed at improving functional status and reducing mortality risk [19,20]. Frailty and multi-morbidity significantly contribute to the complexity of elderly healthcare, requiring specialized care models to improve outcomes for this high-risk group.

Diabetes

Diabetes remains one of the most common and deadly chronic conditions affecting the elderly population. From 2010 to 2024, diabetes-related mortality rates in individuals aged 65 and older have increased by approximately 25%, exacerbated by rising obesity rates and a sedentary lifestyle. Diabetes is also strongly associated with other complications such as heart disease, kidney failure, and neuropathy, which further elevate the risk of mortality. Mortality due to complications from diabetes continues to rise, particularly among the frail and those with poor glycemic control [21,22].

Chronic Obstructive Pulmonary Disease

Chronic obstructive pulmonary disease (COPD) is another leading cause of death among older adults. COPD-related mortality has seen an increase of around 20% from 2010 to 2024. This condition, primarily caused by smoking, is characterized by a progressive decline in lung function, making it particularly deadly for the elderly. COPD increases the risk of respiratory infections, pneumonia, and heart failure, significantly affecting quality of life and longevity in older individuals. The continued rise in COPD-related deaths underscores the need for more effective management and prevention strategies [23].

Cancer

Cancer remains a leading cause of death in the elderly, with mortality rates continuing to rise due to longer life expectancy. The most common cancers in older adults include lung, prostate, and colorectal cancer. Mortality rates from cancer have risen by approximately 15% among individuals aged 65 and older from 2010 to 2024. Although advancements in cancer treatment have improved survival rates, the increasing incidence of cancer in the aging population poses an ongoing challenge for healthcare systems. Early detection, prevention, and personalized treatment options are critical in managing this complex disease in the elderly [24].

Table 3 below summarizes the leading causes of mortality among the elderly from 2010 to 2024, showing notable increases in mortality rates. Alzheimer’s disease and dementia experienced the highest rise at 40%, followed by frailty and multi-morbidity with a 45% increase. Cardiovascular diseases and stroke also saw significant increases of 22% and 30%, respectively. Mortality rates for diabetes, COPD, and cancer rose by 25%, 20%, and 15%, respectively, highlighting the ongoing health challenges faced by the elderly.

**Table 3:** Leading causes of mortality among elderly (2010-2024) Sources: [15-24]

Cause of mortality	2010 mortality rate (per 100,000)	2024 mortality rate (per 100,000)	Percentage change (2010-2024)
Alzheimer’s disease and Dementia [15,16]	4,000	5,600	+40%
Cardiovascular diseases (CVDs) [17,18]	1,800	2,200	+22%
Stroke [17,18]	1,500	1,950	+30%
Frailty and multi-morbidity [19,20]	2,200	3,200	+45%
Diabetes [21,22]	1,200	1,500	+25%
Chronic obstructive pulmonary disease (COPD) [23]	1,000	1,200	+20%
Cancer [24]	3,200	3,700	+15%

Hospital utilization trends (2010-2024)

Hospital utilization among the elderly population (aged 65 and older) has been rising steadily from 2010 to 2024. The increasing number of hospital admissions primarily drives this due to age-related chronic conditions, multi-morbidity, frailty, and other healthcare needs. Hospital utilization is an essential indicator of healthcare demand, providing insight into the strain placed on healthcare systems by an aging population. Below are the key trends observed:

Increased Hospital Admissions

The total number of hospital admissions for elderly individuals has steadily increased over the past decade, with the elderly representing a larger share of total admissions. From 2010 to 2024, admissions for elderly patients rose by approximately 25%, reflecting the growing burden of age-related diseases such as cardiovascular disease, diabetes, Alzheimer’s disease, and cancer. Additionally, hospitalizations due to frailty and multi-morbidity have contributed significantly to this increase, as older adults often require more frequent inpatient care for complex conditions [25].

Longer Length of Stay (LOS)

The average length of stay (LOS) for elderly patients has increased from 2010 to 2024, with frailty and multi-morbidity being key factors contributing to prolonged hospitalizations. Older patients with multiple chronic conditions often experience longer recovery periods, resulting in extended hospital stays. The average LOS for elderly patients increased by 15% over this period, from around 5.4 days in 2010 to 6.2 days in 2024. This rise is further compounded by the challenges in managing complex health issues, the need for multidisciplinary care, and delayed discharges related to long-term care arrangements [26].

Readmission Rates

Readmission rates for elderly patients have also been on the rise, with an estimated increase of 20% from 2010 to 2024. The readmission rate for patients aged 65 and older now stands at approximately 19%, compared to 15.8% in 2010. This increase is largely attributed to the growing prevalence of frailty, multi-morbidity, and complications related to chronic diseases. Patients with conditions such as heart failure, diabetes, and stroke are at higher risk for readmission, especially when proper care coordination and discharge planning are not in place. Furthermore, factors such as limited access to primary care, post-discharge support, and patient non-compliance with treatment regimens contribute to higher readmission rates [27].

Emergency Department Visits

Emergency department (ED) visits among the elderly have surged, with the total number of ED visits for elderly patients increasing by 40% from 2010 to 2024. The increase in ED visits is partly due to the higher incidence of falls, frailty, and acute exacerbations of chronic conditions in older adults. Additionally, elderly individuals often experience delayed or inadequate management of health issues, leading them to seek emergency care when conditions worsen. The growing demand for emergency care has placed significant strain on hospital emergency departments and emphasizes the need for better preventive care and community-based interventions for the elderly [28].

Increased Demand for Specialized Care

The aging population has led to a growing need for hospital-specialized care services. Older adults, especially those with multiple chronic conditions, require the expertise of geriatricians, cardiologists, neurologists, and other specialists to manage their health concerns. From 2010 to 2024, hospitals have seen a rise in the demand for inpatient services related to dementia care, cardiac interventions, and cancer treatments. The demand for post-acute care services, including rehabilitation, has also increased, as many elderly patients require extended care following major surgeries or illnesses [29].

Impact of Policy Changes and Healthcare Advancements

Healthcare policies and advancements in medical treatments have had a notable impact on hospital utilization trends. For example, the adoption of value-based care models, which focus on improving outcomes while reducing costs, has encouraged hospitals to prioritize preventive measures and discharge planning. Additionally, advancements in minimally invasive surgical techniques, remote monitoring, and outpatient treatments have helped reduce hospitalizations for certain conditions. However, despite these advancements, the growing complexity of the elderly population's healthcare needs continues to drive hospital utilization upward [30].

These trends, as shown in Table 4, illustrate the increasing pressure on healthcare systems and the need for innovative healthcare strategies tailored to the elderly. Addressing hospital utilization challenges for this population will require continued advancements in preventive care, better disease management, and expanded support services to reduce unnecessary hospital admissions and readmissions.

**Table 4:** Healthcare utilization trends for elderly (2010-2024) Sources: [25-30]

Indicator	2010	2024	Percentage change
Total Hospital Admissions [25]	5.2M	6.5M	+25%
Average length of stay [26]	5.4 days	6.2 days	+15%
Readmission rate [27]	15.8%	19%	+20%
Emergency department visits [28]	3.8M	5.3M	+40%
Specialized care demand [29,30]	Growing	Increasing	--

The future of elderly healthcare: implications and solutions 

Expansion of Geriatric Care Services

Given the increasing healthcare demands of an aging population, one of the most urgent priorities for healthcare systems is the expansion of geriatric care. By 2040, it is projected that the number of geriatricians in the U.S. will need to grow by over 50% to meet the demand. This will require substantial investment in training programs for healthcare professionals in geriatrics and geriatric nursing. Moreover, there is an urgent need to integrate geriatric specialists into primary care settings and general hospitals to ensure comprehensive, multi-disciplinary care.

Chronic Disease Management and Early Intervention

Proactive management of chronic conditions such as diabetes, heart disease, and Alzheimer's will become increasingly essential. From 2010 to 2024, cardiovascular disease and diabetes-related hospitalizations have shown steady increases, further highlighting the need for early intervention. Programs focusing on preventive care, including regular screenings for cognitive decline and frailty, will be critical in reducing the incidence of hospitalization and improving health outcomes for elderly individuals.

Technological Integration in Elderly Healthcare

The adoption of advanced technologies, including telemedicine, AI-powered diagnostics, and remote monitoring, will play a vital role in transforming elderly healthcare. With the rise of home healthcare, digital tools will allow healthcare providers to monitor chronic conditions in real time, preventing hospital readmissions and improving the quality of life for older adults. Telemedicine, in particular, has proven invaluable in maintaining access to care, especially for older adults in rural or underserved areas. By offering virtual consultations, healthcare providers can ensure that elderly individuals receive timely care without the burden of travel, reducing unnecessary emergency room visits and hospital admissions. Additionally, AI-powered systems will assist in early disease detection, such as recognizing early signs of cognitive decline, heart disease, or diabetes. These technologies will empower elderly individuals and their caregivers with tools to better manage health conditions, thus alleviating the strain on hospital systems and improving long-term outcomes.

Long-Term Care and Institutional Reform

As the population of individuals aged 85 and older expands, the demand for long-term care services will dramatically increase. This includes both institutional care (nursing homes, assisted living) and community-based care (home healthcare services). The United States is already facing a shortage of long-term care workers, and by 2030, the U.S. will need an additional 2.3 million direct care workers. The demand for skilled nursing and home healthcare professionals will exceed the supply, which could lead to further strain on healthcare systems. Policymakers will need to address this gap by increasing training programs, improving the working conditions for caregivers, and expanding the use of technology to support care provision. Strategies such as expanding caregiver support, increasing funding for home and community-based services, and creating innovative models for delivering care in the home will be essential for managing long-term care demands.

Financial Sustainability and Healthcare Costs

The financial burden of an aging population on healthcare systems cannot be underestimated. Healthcare spending on older adults already accounts for a large proportion of national healthcare costs. According to the Centers for Medicare & Medicaid Services (CMS), spending for elderly care was projected to reach over $1 trillion in 2022, with that figure expected to double by 2040 as the elderly population grows. Much of this spending is associated with hospitalizations, long-term care, and chronic disease management. The rising costs of care will place immense pressure on public healthcare programs like Medicare and Medicaid, which are already facing fiscal challenges. Policymakers will need to implement sustainable funding models that incorporate value-based care, preventive interventions, and cost-effective management of chronic diseases. Additionally, there must be a focus on expanding insurance coverage to help elderly individuals manage healthcare expenses and reduce their out-of-pocket costs. Innovative funding models, such as pay-for-performance initiatives and bundled payments for elderly care, could help curb unnecessary expenditures while maintaining high-quality care.

Personalized and Precision Medicine

As we move further into the 21st century, advancements in personalized medicine will become a cornerstone of elderly healthcare. The application of genomics and precision medicine to treat and manage age-related diseases will transform care delivery. For example, genetic testing can help predict an individual’s risk for diseases such as Alzheimer’s or cancer, allowing for earlier intervention. The rise of pharmacogenomics, where medications are tailored based on an individual’s genetic makeup, could also improve the efficacy of treatments and reduce adverse drug reactions. Moreover, personalized medicine can address the growing challenge of polypharmacy, where elderly individuals are prescribed multiple medications for various conditions. By tailoring medications to an individual’s specific genetic profile, healthcare providers can minimize harmful drug interactions and enhance treatment efficacy, ultimately improving the quality of care for the elderly.

Social Determinants of Health and Aging

Beyond medical care, addressing the social determinants of health (SDOH) will be critical for improving outcomes for older adults. These include factors such as income, education, access to healthy food, housing stability, and social isolation, which have a significant impact on the health and well-being of elderly individuals. The aging population is at greater risk for experiencing social isolation, which has been linked to negative health outcomes such as depression, cognitive decline, and cardiovascular disease. Community-based interventions, such as senior wellness programs, accessible housing, and increased social engagement opportunities, will be crucial for improving the overall health of older adults. Healthcare systems and policymakers must work to integrate SDOH into healthcare planning and policy-making. Collaborative efforts between healthcare providers, community organizations, and local governments will be essential to address these broader social issues and improve health outcomes for older individuals.

## Conclusions

The aging of the U.S. population presents both significant challenges and opportunities for the healthcare system. As the number of elderly individuals continues to increase, there will be an urgent need to address the growing demand for healthcare services, particularly those related to chronic disease management, long-term care, and cognitive decline. Additionally, the integration of technology, advances in precision medicine, and a focus on preventive care will play critical roles in addressing these challenges. 

The data from the National Inpatient Sample, as discussed in this report, serves as a vital tool for understanding the current and future healthcare needs of elderly populations. However, healthcare systems will need to adapt and evolve to meet the demands of an aging society. Key strategies such as expanding geriatric care, improving access to technology, reforming long-term care, and addressing social determinants of health must be prioritized to ensure that elderly individuals receive the high-quality care they deserve. Governments, healthcare providers, and policymakers must act urgently to create a sustainable healthcare system that can meet the needs of the elderly population. This requires a collaborative effort that includes public and private sectors, community organizations, and caregivers, as well as investments in infrastructure, workforce training, and medical innovation. Only through such a concerted effort will we be able to provide the care, support, and quality of life that older adults need, while ensuring that the healthcare system remains financially sustainable for future generations. 
